# Wi-Fi and Satellite-Based Location Techniques for Intelligent Agricultural Machinery Controlled by a Human Operator

**DOI:** 10.3390/s141019767

**Published:** 2014-10-22

**Authors:** Domagoj Drenjanac, Slobodanka Tomic, Juan Agüera, Manuel Perez-Ruiz

**Affiliations:** 1 The Telecommunications Research Center Vienna (FTW), Vienna 1220, Austria; E-Mails: drenjanac@tfw.at (D.D.); tomic@ftw.at (S.T.); 2 Universidad de Córdoba, Área de Mecanización y Tecnología Rural, Dpto. de Ingeniería Rural. Córdoba 14005, Spain; E-Mail: jaguera@uco.es; 3 Aerospace Engineering and Fluid Mechanics Department, University of Seville, Ctra. Sevilla-Utrera km 1, Seville 41013, Spain

**Keywords:** DGNSS, autonomous vehicle, RTK-GNSS, trilateration

## Abstract

In the new agricultural scenarios, the interaction between autonomous tractors and a human operator is important when they jointly perform a task. Obtaining and exchanging accurate localization information between autonomous tractors and the human operator, working as a team, is a critical to maintaining safety, synchronization, and efficiency during the execution of a mission. An advanced localization system for both entities involved in the joint work, *i.e.*, the autonomous tractors and the human operator, provides a basis for meeting the task requirements. In this paper, different localization techniques for a human operator and an autonomous tractor in a field environment were tested. First, we compared the localization performances of two global navigation satellite systems’ (GNSS) receivers carried by the human operator: (1) an internal GNSS receiver built into a handheld device; and (2) an external DGNSS receiver with centimeter-level accuracy. To investigate autonomous tractor localization, a real-time kinematic (RTK)-based localization system installed on autonomous tractor developed for agricultural applications was evaluated. Finally, a hybrid localization approach, which combines distance estimates obtained using a wireless scheme with the position of an autonomous tractor obtained using an RTK-GNSS system, is proposed. The hybrid solution is intended for user localization in unstructured environments in which the GNSS signal is obstructed. The hybrid localization approach has two components: (1) a localization algorithm based on the received signal strength indication (RSSI) from the wireless environment; and (2) the acquisition of the tractor RTK coordinates when the human operator is near the tractor. In five RSSI tests, the best result achieved was an average localization error of 4 m. In tests of real-time position correction between rows, RMS error of 2.4 cm demonstrated that the passes were straight, as was desired for the autonomous tractor. From these preliminary results, future work will address the use of autonomous tractor localization in the hybrid localization approach.

## 1. Introduction

With the advent of semiconductor technology and the associated development of embedded controls and sensing technologies, agricultural equipment manufacturers have focused on reducing the role of the human operator in the control loop. Satellite-based localization approaches have become matured, and numerous applications in agriculture and forestry in developed countries have taken advantage of the centimeter-level accuracy and precision of global navigation satellite systems (GNSS) receivers. GNSS receivers are a key element in precision agriculture technologies (e.g., precision planting systems) and autonomous agricultural vehicles (e.g., system that integrate inertial sensors with GNSS capability for vehicle automation) because position information is a prerequisite for site-specific crop management [1,2].

State-of-the-art robotic fleet management is advancing modern precision agriculture towards the goal of the complete automation of agricultural and forestry processes [3]. To guarantee the achievement of high mission standards, autonomous vehicle control is combined with complementary human operator interaction. The main requirement of this interaction is to enable a human operator to configure, monitor, detect and diagnose potential and existing problems and faults in the system and to resolve them so that the system can proceed with the mission execution [4]. However, intelligent agricultural machinery also requires sophisticated hardware and software to adapt to changing environments and accomplish demanding tasks in a safe and efficient manner. Two navigation approaches have become essential for intelligent vehicles systems: combining local information with global localization to enhance autonomous navigation and integrating inertial system information with real-time kinematic (RTK)-GNSS data for vehicle automation [2,5].

Innovative robotic research and applications have enabled robots to act in direct support of and interact with a human partner. As a result, humans and robots are coupled in time and space during the execution of agricultural operations. This type of joint collaboration requires accurate positioning mechanisms to preserve human safety and maintain mission efficiency. Therefore, this paper focuses on providing high-end localization techniques for human operator and intelligent off-road vehicles in a precision agriculture scenario.

Providing an accurate system would require the human operator to carry a large system containing a special antenna, an external GNSS receiver and a battery, which could be difficult to handle and would increase the cost of the system. Thus, our goal is to create a low-cost solution. The former approach is common and well known in outdoor applications, e.g., in various types of robot navigation [6]. The latter approach provides localization capability in indoor wireless-supported environments [7].

Because the robots in our agricultural scenario are equipped with high-quality wireless routers, it is feasible to use network information for positioning purposes. Due to the poor distance estimates produced by the use of radio signals for measuring the distance between nodes [8], we focus on the objective of providing a hybrid localization solution that combines distance estimates and RTK localization of an autonomous tractor in our scenario. Therefore, we introduce a hybrid localization approach that has two components: (1) a localization algorithm based on the received signal strength indication (RSSI) from the wireless environment and (2) the acquisition of autonomous tractor RTK coordinates when the human operator is nearby [9]. However, this hybrid approach has not been conceived to be a standalone positioning solution; instead, it has been planned as a positioning mechanism to complement the existing GNSS receiver.

The remainder of this paper is structured as follows. Section 2 describes the materials and methods used to localize the human operator and intelligent off-road vehicle in the relevant scenario. The second section concludes with a description of field experiments performed to test the localization of the human operator and intelligent off-road vehicle. Section 3 discusses the results of the two experiments. Finally, Section 4 concludes the paper and describes future work.

## 2. Materials and Methods

This section describes two experiments conducted in the precision farming scenario within the Robot Fleets for Highly Effective Agriculture and Forestry Management (RHEA) project. The first experiment tested two localization techniques for locating a human operator in a dynamic and unstructured environment based on GNSS and RSSI. The second experiment evaluated an RTK-based positioning system deployed on each intelligent vehicle.

### 2.1. Precision Farming as a Motivating Scenario

The precise management of agricultural land has been made possible due to the availability of new technologies such as GNSS, geographic information systems (GIS), sensors, automated agricultural machinery, and high-resolution imaging. Precision in the application of agricultural chemicals can improve crop quality, increase human safety, and reduce production costs. In the RHEA project, sustainable crop management is achieved via fleet of heterogeneous (*i.e.*, land-based and aerial), intelligent vehicles equipped with advanced sensors and actuators. Precise application can only be achieved using advanced localization equipment and techniques. Because this study focuses on the navigation in an agriculture area, coordinate height components were not considered; however, this component is crucial in geodetic applications.

In the RHEA scenario, the human operator controls an autonomous vehicle using a user portable device (UPD) that has a built-in low-cost localization module. The low-cost GNSS localization modules are sufficient for localizing the UPD in a clear field, similar to the RHEA scenario, with a direct line of sight (LOS) and a reduced multipath effect. However, most agricultural work is performed in fields either near forests or in forests, where shadowing due to dense foliage attenuates the direct signal. The positioning capabilities of the UPD can be enhanced by providing an external GNSS positioning system, in which the user must carry a larger system containing a special antenna, an external receiver and a battery, however, the system could be difficult to handle. Therefore, the proposed hybrid solution could be utilized in GNSS inaccessible environments, such as dense forests, where agricultural machines are used for tree harvesting or similar work.

### 2.2. Localization of the Human Operator

Tests were conducted to learn the trade-offs between UPD localization approaches that aggregated data from an internal low-cost GNSS system, the wireless network signal strength, and an external DGNSS receiver with an EGNOS correction signal. The proposed hybrid approach is based on combining data from two sources: (1) satellite-based positioning and (2) positioning based on the RSSI from the wireless routers used in the RHEA scenario.

#### 2.2.1. User Portable Device and GNSS Receivers Specifications

The user portable device (UPD) is a tablet PC with remote control functions for the RHEA intelligent vehicles. Due to the challenges encountered in an outdoor environment, a rugged tablet PC (IP 65) was selected as the implementation platform, equipped with an Intel Atom Z530 1.6 GHz processor, 2 GB of DDR2 RAM, and 64 GB of SSD disk space. This device has a 7” touch screen, an 802.11b/g/n wireless adapter and an integrated GPS receiver and runs the Windows 7 operating system.

Internal and external GNSS receiver modules were used to determine the two-dimensional (2D) position (*i.e.*, the latitude and longitude) of a user during localization tests. The internal GNSS module (model FV-M9, San Jose Technology, Inc., Tucheng, Taiwan) supports four types of differential correction signals: WAAS within North America, EGNOS within Europe, GAGAN in South Asia and MSAS within Japan and Southeast Asia. It communicates with the appropriate geostationary satellites, which provide a regional correction signal and determine the location of the user in the field. According to its specifications, its position accuracy is approximately 3.3 m. The external DGNSS module (model AgGPS 162, Trimble Navigation Ltd., Sunnyvale, CA, USA) is a combined antenna and GNSS L1 receiver capable of satellite-based augmentation system (SBAS) navigation using WAAS, EGNOS and MSAS. The combination of a rugged housing and SBAS accuracy make this equipment suitable and economical for a wide range of agricultural applications, such as yield monitoring, field mapping and guidance systems.

#### 2.2.2. Using a Wireless Network for Localization

**Figure 1.**
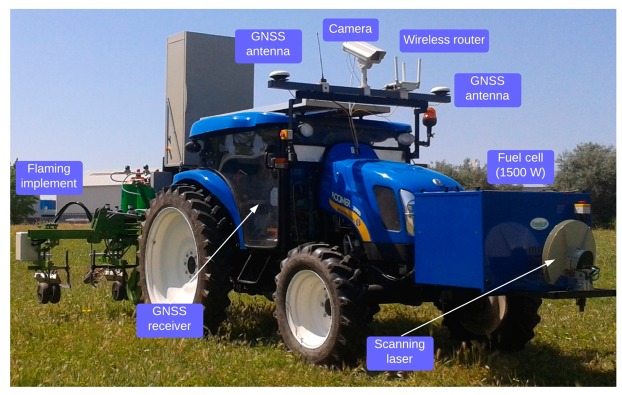
Intelligent off-road vehicle unit configuration.

Each autonomous tractor in the RHEA scenario is equipped with an RTK positioning system that provides an accurate position for the wireless node installed on the vehicle (Figure 1). Therefore, we propose a hybrid positioning approach that runs on the UPD and combines the output of an RSSI-based localization algorithm using signals from the wireless routers mounted on the autonomous tractors and a procedure for the acquisition of the tractor coordinates when a user carrying the UPD is near the tractor. This hybrid approach is conceived as a complementary positioning mechanism to improve the accuracy of the UPD position provided by the low-cost GPS receiver integrated into the UPD. The first step to realizing the hybrid solution was the implementation of the RSSI-based localization algorithm. This technique provided insight into the performance of the radio-based positioning solution. In the second step, the implemented solution was integrated with a positioning procedure that will be activated when a user is near the autonomous tractor. Due to limited time, the development of this procedure is planned as future work. The subsystems necessary for the realization of the network-based localization algorithm are channel characterization and localization algorithms based on radio channel data.

##### Channel Characterization

The goal of the channel characterization process was to determine a suitable radio channel model for the wireless environment. To model the channel within the RHEA scenario, range tests were performed and numerous RSSI measurements were collected at different distances between the receiver (*i.e.*, the UPD) and the transmitter (*i.e.*, the wireless router mounted on the autonomous tractor). Because the UPD operates in a dynamic environment in which the formation of the autonomous vehicles often changes, the communication channel model accounts for the orientation of the UPD relative to the router. The measurements indicated that the RSSI performance was influenced by the UPD orientation relative to the wireless router (e.g., whether the UPD was facing the router or the user had turned his back to the router).

##### Localization Algorithms

Two localization algorithms requiring only a limited number of reference nodes were implemented. Reference nodes are also known as beacon nodes because they have accurate information about their accurate locations and are thus used as reference points for the localization of other nodes. For this paper, two localization algorithms were considered and implemented: multilateration and min-max [10].

Multilateration is a simple range-based, decentralized localization algorithm based on geometric principles. To estimate its position, a node collects beacon messages and estimates its distance to each beacon. The node then computes its position by determining the intersection of the circles centered at the positions occupied by the beacons with radii equal to the estimated distance between the beacons and the node [6]. The intersection would ideally be a single point, but the intersection typically identifies an area in which the node is located due to accumulated error.

Min-max is a popular localization algorithm due to its simple implementation. To estimate its position, a node creates an association between each beacon position and the distance to that beacon. The node then creates pairs of horizontal and vertical lines around each beacon such that the minimum distance between each line and the beacon position equals the estimated node-beacon distance. The node locates itself in the center of the rectangular area between the innermost horizontal and vertical lines [6]. The localization error depends on the size of the rectangular area. A smaller intersection enables better localization.

### 2.3. Autonomous Tractor Localization

An autonomous agricultural tractor was designed and constructed under a European research project and is part of a 3-unit fleet of similar vehicles [11]. The platform for autonomous vehicle was a conventional 38 kW tractor (New Holland model Boomer T3050, 3-point hitch, Zedelgem, Belgium) that was retrofitted for autonomous agricultural operations. Figure 1 shows the equipment configuration used in the field experiments.

A specially fabricated frame was attached to the tractor and used to mount the necessary equipment, including the on-board computers, the inertial measurement unit, modems for navigation and connector boxes. The motion of the autonomous tractor had three primary degrees of freedom (longitudinal, lateral and yaw). The tractor controller was responsible for sensing the vehicle location and heading angle.

To create a fully autonomous agricultural system capable of ensuring precise navigation, it is necessary to configure a hardware and software framework that merges perception (*i.e.*, accurate vehicle positioning) and action (*i.e.*, steering and speed control). The hardware framework should be modular, flexible and robust, including real-time multitasking capabilities and integrating modern standard communication protocols. Specifically, the vehicle controller used in this part of the experiment was based on a cRIO 9082 NI computer, and the control algorithms were developed using the LabVIEW graphical programming environment [9].

**Figure 2.**
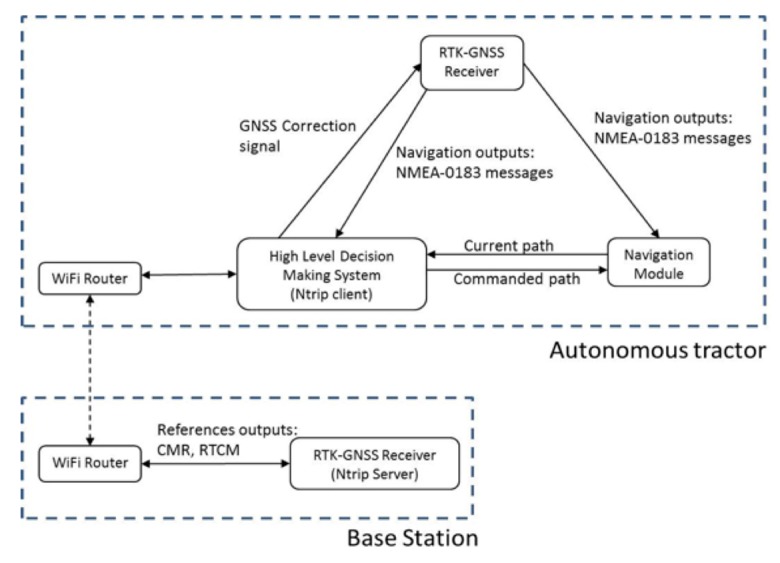
Flowchart of the autonomous tractor localization system.

Figure 2 shows how the GNSS correction signals were captured and transmitted to the receiver through an external port.

### 2.4. Field Experiments

This section describes two experimental testbeds, one for testing UPD localization techniques and the other for testing the RTK positioning system used on the autonomous tractor. Field tests for both the GNSS and RSSI UPD localization techniques were performed during autumn of 2013 at the FTW field experiment site in the Danube Park (latitude: 48°14′16.0″ N, longitude: 16°44′29.6″ E). RTK field tests were performed over a one-week period during the winter of 2013 at the Center for Automation and Robotics field experiment site at the Spanish National Research Council (CISC) in Madrid (latitude: 40°18′48.9″ N, longitude: 03°28′54.8″ W).

#### 2.4.1. GNSS Test Platform

Two testbeds were designed for testing the internal GNSS and external DGNSS receivers. The first testbed was built for static positioning tests in which the receiver maintained a fixed position. Because the user in the RHEA scenario must supervise the entire robotic fleet, the user’s position will change often and will remain constant for a variable amount of time. Thus, we were interested in how the positioning accuracy changed with the time that the user stayed at a single location. Therefore, tests were performed at three locations at which the user stayed 1, 2 and 5 min. The user’s position was tested at the same locations with both receivers.

**Figure 3.**
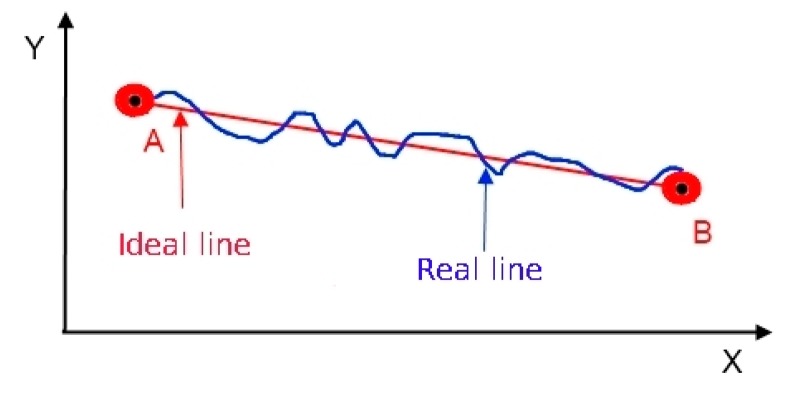
Configuration for measuring errors between the ideal and actual paths.

**Figure 4.**
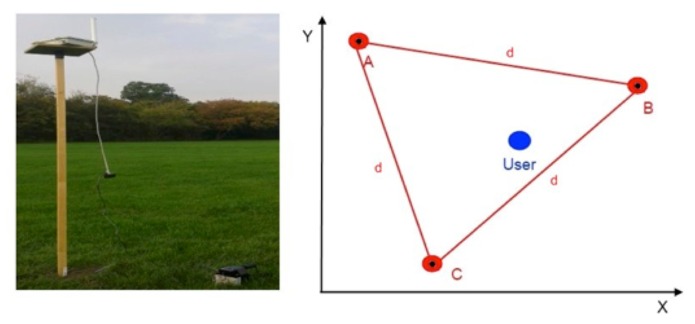
Wireless router on a pole (simulating an autonomous vehicle) and the experimental configuration.

In the second set of tests, positioning errors were calculated from the coordinates of the user’s position along an ideal straight path (Figure 3). First, a straight line was marked on the ground (the ideal line) by stretching a 20 m rope between points (A and B). The GNSS average coordinates for reference points A and B were determined from a GNSS antenna mounted on a 2 m survey pole for 10 min (Figure 4). After the coordinates were determined, the user walked from point A to point B along the stretched rope twice, once carrying the internal GNSS module and once carrying the external DGNSS module. The results from the two modules were compared based on the error between the straight line and the actual trajectory. In both cases, the measurements were sampled at a frequency of 1 Hz.

#### 2.4.2. RSSI Test Platform

The RSSI localization algorithm has two phases: (1) channel modeling based on the sampled data; and (2) the evaluation of the localization accuracy performed using the channel data. Theoretically, the channel modeling process only needs to be performed once, but because the radio channel is unpredictable, it is not unusual to sample the data repeatedly to improve the channel model. The localization algorithm uses the distance to the known nodes determined during the channel modeling phase.

##### Channel Modeling

Range tests were performed at six orientations between the UPD and router to determine the variation of the signal strength with the UPD position. In the first three tests, the user faced the router and set the inclination of the UPD held in his hands to −30°, 0°, and +30°. In the fourth test, the user turned his back towards the router, and in the last two tests, either his left or right side faced the router. The data were sampled at a frequency of 0.5 Hz for 60 s at distances from the router of 5, 10, 20, and 40 m. During the processing of the results, the test during which the user turned his back toward the router were omitted because the signal strength during these tests varied substantially, and the signal was 20% weaker than in the other positions. However, in RHEA, the user supervises an entire mission, which means facing at least one autonomous vehicle and frequently changing his direction of focus.

##### RSSI Localization

The experimental testbed for the RSSI localization algorithm consisted of three configurable wireless routers (Figure 4) transmitting in the 2.4 and 5 GHz spectra. During the localization tests, the routers were configured to transmit in the 2.4 GHz spectrum with a transmission power of 6 dBm. On the receiver side, the user carried the UPD with an integrated wireless card able to scan the available wireless networks and determine their signal strengths. Figure 4 illustrates the experimental configuration, in which three routers formed an equilateral triangle ABC with edge lengths of d = 20 m. The user was located inside the triangle at an unknown position, shown as the blue dot in Figure 4. The wireless router on the left side (Figure 4) was mounted on a wooden pole at a height of 2 m and was powered by an external battery placed on the ground.

Three tests were performed using the testbed during which the user’s orientation with respect to the routers was varied. The first set of localization tests was performed with the user and the UPD facing the router A (Figure 4). In the second set of tests, the user faced router B, and in the last test, the user faced router C. The tests were designed in this manner because the user always faces at least one autonomous tractor. Both the multilateration and min-max localization algorithms were employed simultaneously during each test. The tests lasted 120 s, and the data were sampled at a rate of 0.5 Hz. The collected results were processed and are presented in the next section.

#### 2.4.3. Field Test of the Intelligent Off-Road Vehicle

The three criteria used for choosing the test plot were the following: (1) the plot was nearly flat; (2) the plot was sufficiently large for five 20 m rows; and (3) the plot was within range of the base station used in the experiment.

A static test was performed on top of an approximately 20 m-long building with an open sky. As a dynamic test, the correction signal was measured for 30 min at different times of days on three days within the same week. According to the manufacturer [12], this testing procedure would provide exposure to a sufficient range of satellite constellation to estimate the GNSS system accuracy.

**Figure 5.**
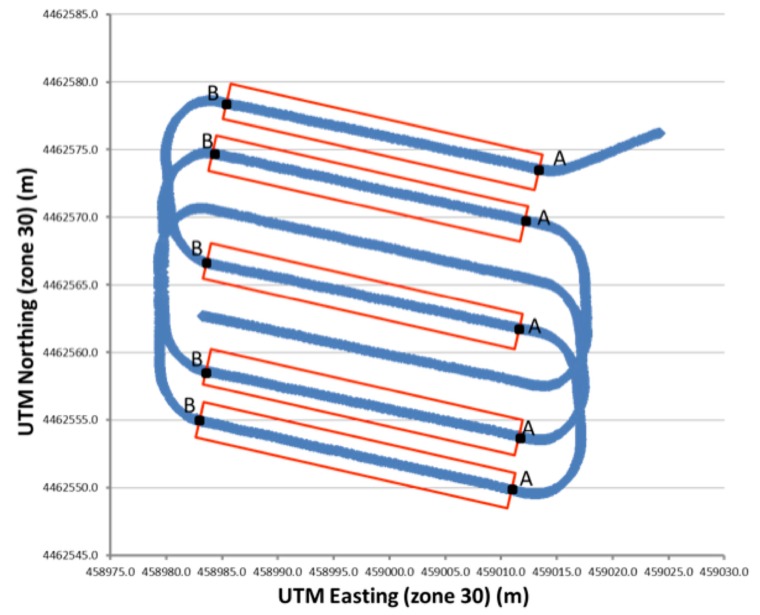
Straight-line path for the autonomous vehicle.

Each dynamic test consisted of five 20 m passes following straight-line paths (Figure 5). The geospatial location of two points (A and B) at both ends of each straight line was determined with an RTK-GNSS receiver using a handheld surveying system interfaced to an RTK-GNSS rover (Trimble model Bx982). The GNSS coordinates for points A and B were obtained by placing the bottom of a 2 m survey pole with a GPS antenna attached at the top on the soil surface and holding the pole vertically using the aid of a bubble level. A WGS 84 Datum was used during all the RTK-GNSS measurements. The position of points A and B were obtained for two purposes: (1) to establish the straight line paths for the autonomous tractor and (2) to mark straight lines on the ground. All passes were traversed at a speed of 2.5 km h^−1^. In this experiment, the RTK-GNSS signal was provided by the base station.

The following raw GNSS data were recorded for all the dynamic tests performed with the autonomous tractor: the UTC time, longitude, latitude, height, velocity, quality of GNSS, PDOP, heading and number of satellites. Only the time, longitude, latitude and heading were used in the accuracy analysis. A program implemented in LabVIEW (National Instruments, Austin, TX, USA) was used to convert the geographic data into UTM coordinates. To determine the accuracy of the intelligent off-road vehicle path with respect to the prescribed path, the single-point cross-track error (XTE) was used, which is defined as the perpendicular distance along the ground from the straight line AB to each error measurement. Measurements were taken every 0.2 m within the ideal straight line path. The total XTE was calculated using the root mean squared (RMS) error of all the single-point XTEs along the length of the straight line [13]. The cross-track error is a useful value that measures the potential skip or overlap between passes of an agricultural vehicle through a field.

For each pass, the RMS error was calculated using the following equations:

(1) $$\varepsilon_{{\text{RMS}}_{t}}=\sqrt{\frac{1}{N_{t}}\overset{N_{t}}{\underset{i=1}{\text{∑}}}e_{it}^{2}}$$

(2) $${e^{2}}_{it}={\left(x_{i}-x_{t}\right)}^{2}+{\left(y_{i}-y_{t}\right)}^{2}$$

where *RMS_t_* is the RMS error for the *t* th pass, *N_t_* is the total number of measurement points for the *t* th pass, and *e_it_* is the distance from the point *i* th point to the *t* th straight line path. For the statistical analysis, the errors were calculated for each measurement. The SAS general linear models procedure [14] was used to test for significant differences in the treatment (based on the RTK data) using an ANOVA. Statistics for the GNSS receiver position accuracy values in static tests were calculated using JMP [13].

## 3. Results and Discussion

This section presents and discusses the preliminary results from the UPD and intelligent off-road vehicle localization tests. The UPD localization tests utilized both the GNSS and RSSI localization methods. Fifteen hundred events were recorded in the static and dynamic GNSS localization tests, and approximately 800 events were recorded in the RSSI localization tests. Of the 16,690 events that were recorded in five passes in the vehicle experiment, 610 were used to determine the level of accuracy. A greater amount of data was collected in this study than in previous studies [15,16]; however, this study was limited to a single manufacturer’s implementation of a RTK-GNSS system.

### 3.1. GNSS Localization

The results from the static tests are shown in Table 1. The columns represent the time that the user stayed in the same location and each row presents the data for a receiver. The values in the table are pairs of standard deviations calculated for the easting and northing coordinates. Because the coordinates are in the UTM format, all values are expressed in meters.

The data in Table 1 show the positioning accuracy obtained using the two GNSS modules. The UPD produced an accuracy of 8 cm easting, and 9 cm northing when the external DGNSS module was used for 1 min. The accuracy increased as more time was spent at a particular; e.g., the accuracy was 3 cm easting and 4 cm northing after 5 min. The best accuracy achieved with the internal GNSS receiver was 5 cm easting and 30 cm northing after 5 min. The DGNSS receiver was twice as accurate in the easting coordinate and approximately five times more accurate in the northing coordinate than the internal GNSS receiver.

**Figure 6.**
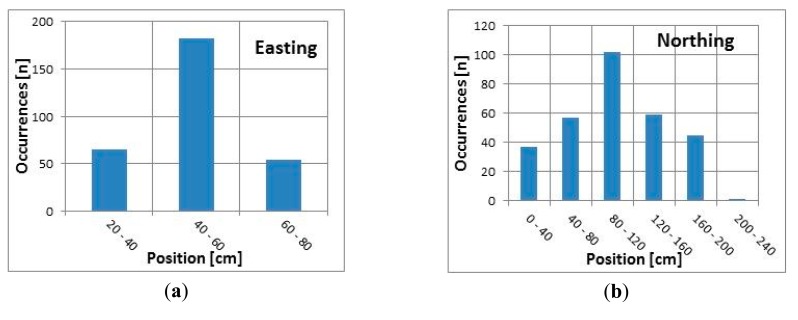
GNSS easting (**a**) and northing (**b**) distribution.

Figure 6a and b present the number of occurrences within each position range measured using the internal GNSS receiver over a 5 min interval (300 samples) for the easting and northing coordinates. All sampled easting locations fall within a range of 60 cm, and approximately 60% of the measurements are within a 20 cm range (40–60 cm, see Figure 6a). All but one recorded that northing locations fall within a range of 200 cm, and approximately 75% of the measurements are within a 120 cm range (40–160 cm, see Figure 6b).

**Figure 7.**
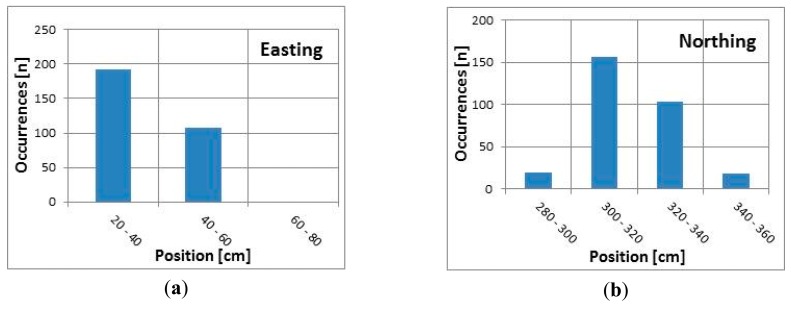
DGNSS easting (**a**) and northing (**b**) distribution.

Figure 7a and b present the number of occurrences within each position range measured using the external DGNSS receiver over a 5 min interval (300 samples) for the easting and northing coordinates. All sampled easting locations fall within a range of 40 cm, and approximately 65% of the measurements are within a 20 cm range (20–40 cm, see Figure 7a). All the sampled values are within the first two intervals (compared to Figure 6a). The recorded northing locations fall within a range of 80 cm, and approximately 90% of the measurements are within 40 cm (300–340 cm, see Figure 7b).

According to the results presented in Table 1 and Figures 6 and 7, both receivers had better easting than northing accuracy. This effect was greater when the internal GNSS receiver was used; the difference between the standard deviations of the easting and northing coordinates was greater than when the external DGNSS receiver was used. However, both receivers provided sub-meter accuracy in all tests. Additionally, there was no dependency between the positioning accuracy and the time spent at one location, or at least no dependency could be found using standard statistical measures, such as the standard deviation.

**Figure 8.**
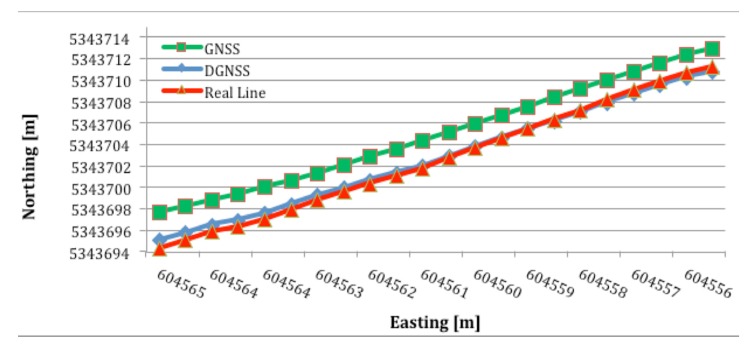
Deviation between the straight line path and the actual path obtained using the GNSS and DGNSS receivers.

Figure 8 present the results from the second set of tests, in which the deviation between the straight line path and actual path was measured. The actual paths for both the internal GNSS and external DGNSS receivers are plotted. There are 22 locations in total, so it took 22 s to cover the 20 m distance between points A and B. The traverse began at the bottom left point and ended at the top right point (*i.e.*, from southeast to northwest).

The red line in Figure 8 represent the straight line path. The other two lines correspond to data from the selected receivers. The blue line, representing the external DGNSS receiver, does not greatly diverge from the straight line compared with the green line, representing the internal GNSS receiver. Moreover, one segment of the blue line—between 604,561 m and 604,557 m—overlaps with the straight line. However, there is no overlap between the straight line and the GNSS data.

The results from Figure 8 are quantified in Table 2. The columns in Table 2 represent two receivers and correspond to the blue and green lines in Figure 8. Table 2 shows the average deviation from the straight line calculated from the set of 22 samples.

Table 2 complements Figure 8 and confirms that the blue line, representing the DGNSS receiver, is much closer to the ideal line (with a 21 cm mean deviation) than the green line, representing the GNSS receiver, which has a 119 cm deviation. Interestingly, the green line has a slope and direction that are similar to the ideal line but shifted by approximately 2 m.

### 3.2. RSSI Localization

The data collected from the channel modeling and RSSI localization tests are presented in this section.

#### 3.2.1. Channel Modeling

**Figure 9.**
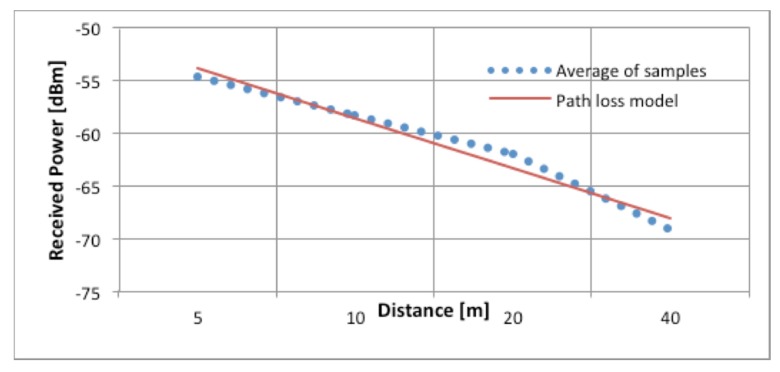
Sampled signal strength values for channel modeling.

The collected test data were statistically analyzed, and the averaged results from the five orientations are plotted in Figure 9 as a function of the distance of the UPD from the router. The blue dotted line in Figure 9 corresponds to the averaged values of all samples collected in the range tests described in Section 2.4.2. The red solid line corresponds to the adapted path loss model, which is based on the free space loss model described in [16].

For this study, the CCIR, HATA, and Walfisch-Ikegami path loss models were compared with the sampled result. However, we considered the free space loss model, described in Equation (3), to be the most suitable because it is possible to adjust the parameter K to best fit the measured values:

(3) $${\text{P}}_{\text{i}}[\text{dBm}]={\text{P}}_{\text{Tx}}+{\text{G}}_{\text{t}}+{\text{G}}_{\text{t}}-\left(\text{K}+20\log_{10}({\text{d}}_{\text{km}})+20\log_{10}({\text{f}}_{\text{MHz}})\right)$$

Equation (3) defines the received signal power *P_i_* (in dBm) at a node *i* placed at a distance *d_km_* from the transmitter as the sum of the transmitted power *P_Tx_* (in dBM) and the transmitter and receiver antenna gains *G_t_* and *G_r_*, respectively, minus the logarithm of the distance *d_km_*, the logarithm of the frequency *f_MHz_*, and a parameter K. We applied the Solver tool in Microsoft Excel to the mean square error to optimize the path loss model parameter K. Compared to the value of K = 32.45 obtained in [17], the optimization process yielded K = 45.35. However, as can be observed in Figure 9, the fit to the experimental results is reasonably good.

Some discrepancies exist between the measured and modeled values at distances between the UPD and the router of less than 10 m. However, these errors should not hinder the performance of the hybrid positioning system due to safety measures that limit the distance between the user and the autonomous tractor to greater than 10 m. When the user needs to perform maintenance on an autonomous tractor, the system can acquire the coordinates of that tractor to determine the user’s current position

#### 3.2.2. RSSI Localization

**Figure 10.**
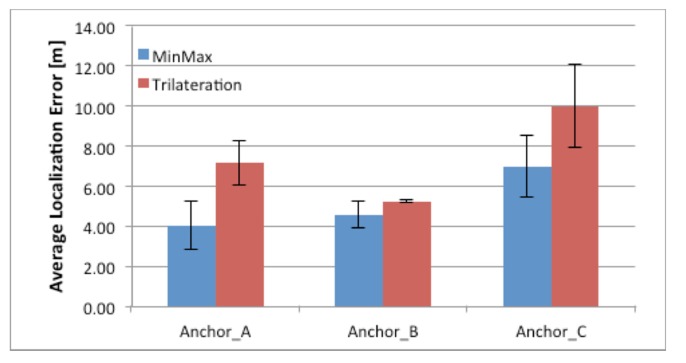
Localization error.

The results of the localization test are presented in Figure 10 which illustrates how the localization error changes with the user orientation.

Anchors A, B, and C correspond to routers A, B, and C, respectively, in Figure 4. The bar values are averages of the sampled results, and each bar includes the standard deviation of the measurements. The red bars represent the localization error calculated by the trilateration algorithm, and blue bars represent the min-max algorithm. Figure 10 shows that the min-max algorithm yields better results and lower localization error than the classic trilateration algorithm. The localization error for the min-max algorithm ranges from 4 m in the best case to 7 m in the worst case. The lowest localization error from the trilateration algorithm is 4.5 m, and the highest is 10 m. The relatively good performance achieved by the min-max algorithm is mainly due to its tendency to locate the user in the center of the area defined by vertical and horizontal lines, as described in Section 2.2.2, using the wireless network for localization.

The lowest localization error and lowest standard deviation were obtained when the user faced router B. This can be explained by examining the testbed geometry in Figure 4. Because the user was facing router B, he had a direct line of sight to it. Moreover, the user had a partial line of sight to router A as well. This finding is due to the position of router B, which is lower on the y-axis than router A. The low localization error in this test case can be attributed to the existence of these lines of sight, which was not the case in the other two tests. If we refer again to the geometry in Figure 4, it can be expected that when the user faces router C, the localization error will be highest because the other two routers (A and B) are behind the user. In conclusion, a high localization error is a consequence of an unpredictable radio channel and a relatively small number of anchors. As observed in [10], the localization error decreases as the number of anchors increases.

The behavior of the three approaches for UPD localization, namely, internal GNSS, external DGNSS, and RSSI, has been analyzed using real data from the RHEA scenario. We have shown that the external DGNSS approach yields better performance than the other approaches. However, external DGNSS is also the most expensive approach and requires the operator to carry external equipment. Moreover, the internal GNSS solution has two significant advantages over the other two approaches: (1) it also offers sub-meter precision and (2) the positioning module is integrated into the portable device and can thus be easily used. Although RSSI localization algorithms are primarily useful for indoor positioning and their positioning accuracy is not comparable to the accuracy provided by GNSS, we decided to test them in a dynamic outdoor environment as a complementary solution to the existing GNSS modules. However, the results showed that RSSI localization produces a substantial positioning error, and thus, the proposed localization technique has substantial limitations when used in the RHEA scenario, in which humans and autonomous vehicles share the same space.

Hutchens *et al.* [18] measured the GNSS localization error in various agricultural environments: an open field, a small forest, and a medium forest. Interestingly, the average localization error in the open field was 1 m, 8 m in a small forest, and nearly 10 m in medium forest. These results encourage the use of a hybrid solution in forests because the achieved localization error is, in some cases, smaller than that reported in [18]. However, two assumptions must be tested for the hybrid method to outperform traditional GNSS localization in forests: (1) anchor nodes, *i.e.*, autonomous tractors, must be able to acquire GNSS signals due to high-end equipment (RTK) and (2) the existing channel model must be adapted to the new environment. Under these two assumptions, the hybrid localization approach may be able to outperform traditional solutions.

### 3.3. RTK-GNSS Dynamic Test

Table 3 shows the RMS and standard deviation values for the error produced by the GNSS receiver mounted on the off-road vehicle when using RTK-GNSS correction signals. The rover receiver had a horizontal accuracy of 2.5 cm and a vertical accuracy of 3.7 cm when used on a continuous real-time basis. This level of accuracy was expected because the RTK technique can determine the sensor position within a few centimeters [19]. The 2.4 cm average for the RMS cross-track error in the RTK correction signal system indicates that the passes were relatively straight, as was desired for the autonomous tractor. Between the rows there was an error with a constant standard deviation; the average of this value for the RTK technique was 1.43 cm. These results demonstrate that the tractor controller was able to track each straight line with a standard deviation of better than 3.5 cm; the vehicle lateral position error never deviated by more than 4 cm when using the RTK technique. Therefore, fully autonomous vehicles could be used for automated precision farming in many other applications, such as site-specific management of weed control in extensive crops, variable-rate application in orchards and vineyards using the appropriate implement, and variable-rate application of fertilizer based on yield maps.

The mounting location of the GNSS antennas on the autonomous tractor provided a clear view of the entire sky. This maximized the potential for obtaining good satellite geometry, and constant RTK-GPS quality was maintained for the recording of all data.

## 4. Conclusions and Future Work

In this paper, we analyzed existing localization techniques for mobile users and autonomous tractors collaborating in unstructured environments while performing complex agricultural tasks. Two well-known approaches for human operator locations, GNSS and DGNSS, and one approach for autonomous tractors, RTK-GNSS, were tested. Although both GNSS and DGNSS approaches yielded satisfactory localization accuracy in the test environment, we want to find an alternative localization system that can operate in GNSS inaccessible environments and thus complement existing techniques. Therefore, our focus was on determining the feasibility of the proposed hybrid localization approach combining RSSI information from wireless routers mounted on the autonomous tractor with RTK coordinates from the tractors. The hybrid solution was planned as a complement to the standard positioning techniques for mobile users. The complementary solution is activated in situations in which the GNSS signal is either poor or lost. Due to poor channel modeling, the RSSI based localization algorithm yielded a substantial positioning error. However, the localization system installed on autonomous tractor had a high accuracy of approximately 2.4 cm. Because the main assumption is that the mobile user will obtain the autonomous tractor coordinates when near the vehicle, a low position error on the vehicle could mitigate errors in the RSSI localization approach. Thus, this approach may improve localization accuracy and may be used in harsh environments where the GNSS signal is severely attenuated due to dense foliage and obstructed LOS.

Because of the implemented localization systems, the RTK on autonomous tractors and the RSSI on the UPD, future work will focus on utilizing the vehicle locations in the hybrid localization approach. Additionally, we will use the fourth wireless router at the base station to increase the number of anchors and will fine-tune the radio channel. After this is accomplished, we expect to achieve lower localization errors that will be acceptable for scenarios in which humans and robots work together.

## Figures and Tables

**Table 1.** Static positioning accuracy.

	**1 min**	**2 min**	**5 min**
**GNSS Δ [E,N] [m]**	[0.13, 0.33]	[0.10, 0.30]	[0.05, 0.30]
**DGNSS Δ [E,N] [m]**	[0.08, 0.09]	[0.04, 0.05]	[0.03, 0.04]

**Table 2.** Average deviations from the straight line path.

	**DGNSS**	**GNSS**
**Mean [m]**	0.21	1.19

**Table 3.** Statistics for the GNSS receiver using RTK correction signals on the autonomous tractor in motion.

**Row**	**Measurements**	**RTK-GNSS (cm)**	

		**RMS**	**S.D.**
1	122	2.71	2.10
2	122	3.36	1.52
3	122	1.36	0.87
4	122	2.93	1.61
5	122	1.65	1.05

All Rows	610.00	2.40	1.43
